# Intermittent Preventive Treatment of Malaria in Pregnancy and the Impact on Neonates in African Countries as Assessed by Entropy Weight and TOPSIS Methods

**DOI:** 10.3390/jcm13206231

**Published:** 2024-10-18

**Authors:** Maria Tzitiridou-Chatzopoulou, Georgia Zournatzidou, Eirini Orovou, Lazaros Lavasidis, Arsenios Tsiotsias, Panagiotis Eskitzis, Dimitrios Papoutsis

**Affiliations:** 1 Department of Midwifery, School of Healthcare Sciences, University of Western Macedonia, Keptse, 50200 Ptolemaida, Greece; mtzitiridou@uowm.gr (M.T.-C.); eorovou@uowm.gr (E.O.); lavasidis@yahoo.gr (L.L.); atsiotsias@uowm.gr (A.T.); peskitzis@uowm.gr (P.E.); dpapoutsis@uowm.gr (D.P.); 2 Department of Business Administration, University of Western Macedonia, 51100 Grevena, Greece

**Keywords:** Intermittent preventive treatment, pregnancy, neonatal outcome, Africa, zinc, ORS

## Abstract

**Background/Objectives**: In regions of Africa with a high prevalence of malaria, pregnant women in their first or second trimester should be administered intermittent preventive treatment in pregnancy (IPTp). However, infants may contract malaria despite the IPTp therapy that their mothers have received. The objective of the present investigation was to assess the symptoms and various treatments for neonatal malaria. **Methods**: Entropy weight and TOPSIS were used to achieve the study goal. The TOPSIS multi-attribute decision-making system was used to assess newborn malaria symptoms and select the optimal treatment, even for mothers receiving IPTp medication during pregnancy. The entropy weight approach calculated TOPSIS attribute weights. The present research used UNICEF data for 14 African nations in 2023. **Results**: The results indicated that neonates whose mothers received IPTp therapy ultimately contracted malaria, with diarrhea being the primary symptom. It is important to note that health providers administer a combination of zinc and oral rehydration solution (ORS) to infants as the most effective treatment for malaria symptoms, thereby abandoning the first-line treatment for malaria, artemisinin-based combination therapy (ACT). **Conclusions**: The most effective treatment for neonatal malaria is a combination of zinc and ORS, although less than half of children in Africa have access to ORS. Therefore, the findings of this study may encourage African countries to prioritize co-pack therapy in their procurement and supply, healthcare provider training, and expenditures. This therapy will also help alleviate the symptoms of malaria in neonates.

## 1. Introduction

Malaria during pregnancy is a significant global health issue that has major impacts on the well-being of both mothers and babies. In 2021, the African region under the jurisdiction of the World Health Organization (WHO) saw a substantial impact of malaria infection, which affected about 13.3 million pregnancies. Therefore, the region experienced a 11% mortality rate among newborns and a 20% mortality rate among fetuses. The World Health Organization (WHO) recommends that women who live in areas with moderate to high malaria transmission rates receive at least three doses of sulfadoxine-pyrimethamine as an intermittent preventive treatment for malaria in pregnancy (IPTp) [1,2]. It is necessary to provide this drug after every routine appointment at a prenatal care clinic, starting in the second trimester of pregnancy. Research has shown that the administration of IPTp decreases the prevalence of low birthweight infants by 29%, acute maternal anemia by 38%, and neonatal mortality by 31%. IPTp is among the few healthcare interventions that have shown an effective reduction in perinatal mortality. Although it is very cost-effective, the extent of coverage it offers remains inadequate [3].

Only a minority of pregnant women living in areas with regular malaria transmission contract symptomatic malaria infections [4,5]. However, these infections are linked to maternal morbidity, which encompasses illnesses like anemia, as well as harmful effects on the child, such as spontaneous abortion, low birth weight, and infant mortality. In areas with low malaria transmission rates, where women of reproductive age lack immunity to the disease, malaria infection in pregnant women can result in anemia, increase the risk of severe malaria, and potentially result in spontaneous abortion, infant mortality, and low birth weight. In areas with high malaria transmission rates, the prevalence of malaria is higher in locations with a significant population of primigravidae [4,6,7]. As the number of pregnancies rises, the incidence of malaria and the parasite load in the blood decrease. Parasite infection is diagnosed using microscopic examination, polymerase chain reaction (PCR) to identify specific nucleic acids, and rapid diagnostic testing utilizing specialist kits to detect plasmodium antigens. Moreover, the WHO strongly supports the use of IPTp with sulfadoxine-pyrimethamine (SP) as a treatment for uncomplicated malaria in pregnant women and children under the age of 5 in the current malaria-endemic areas of Africa, while it recommends the administration of at least three doses of IPTp for malaria to all pregnant women, irrespective of their plasmodial infection status. This is because IPTp has shown efficacy in avoiding maternal anemia and mitigating the likelihood of low birth weight in neonates [8].

Newborns may be more susceptible to malaria because of IPTp, as indicated by observational studies. By fortifying the neonatal immune system to resist the blood-stage of *P. falciparum*, exposure to *P. falciparum* during pregnancy increases the likelihood of *P. falciparum* infection in early life [9,10]. Nevertheless, the possibility of this phenomenon having clinical significance has not been adequately defined. There is a lack of capacity in observational studies on infant malaria and prenatal malaria exposure to incorporate common risk factors that are shared by the mother and neonate, such as postnatal environmental variables or a shared genetic predisposition to higher sensitivity. Therefore, the results of this investigation are restricted. Therefore, it is conceivable that the link between malaria during pregnancy and the risk to infants is due to postnatal exposure to malaria, rather than direct exposure to malaria parasites during gestation. IPTp in pregnancy clinical research offers a distinctive opportunity to investigate whether maternal malaria exposure influences the susceptibility of neonates to malarias. By employing a randomized regimen, this is feasible, as it guarantees that the exposure levels are comparable among all participants [11,12,13].

Thus, the current study aims to evaluate the symptoms and treatment of malaria in infants in fourteen African countries, which are among those with the highest proportion of pregnant women who have received the IPTp therapy, although their infants have contracted malaria and received specific treatment based on the symptoms they have. To approach the research objective of the current study, the two methods of entropy weight and TOPSIS have been used. For the purposes of the study, six criteria have been used that are related to the different symptoms of infants regarding malaria and the treatment they receive from health providers based on these symptoms. The analysis indicates that infants whose mothers received three doses of IPTp therapy during pregnancy contracted malaria, and the symptom that they mostly experienced was diarrhea, which is treated with ORS and zinc therapy. Additionally, one more novel aspect of the current research is that it indicates that febrile infants receiving artemisinin-based combination therapy (ACT) is not a usual case in the countries under investigation. Despite ACT now being generally accepted as the best treatment for uncomplicated falciparum malaria and one of the most effective treatments for infants with malaria, the current study highlights that this is not a panacea.

The structure of the paper is as follows: Section 2 presents the key points of the related literature review in the field under investigation, Section 3 describes the materials and methods used for the analysis, Section 4 presents the results of both entropy weight and TOPSIS analysis, Section 5 discusses the results, implications and limitations of the study, and Section 6 concludes the paper.

## 2. Literature Review

Malaria infection during pregnancy is a significant public health concern. Pregnancy impairs a woman’s immune system, increasing the likelihood of illness, anemia, severe disease, and mortality, and rendering expecting women more susceptible to malaria. The developing child is at increased risk of spontaneous abortion, stillbirth, premature delivery, and low birth weight because of maternal malaria. Infant mortality is substantially affected by low birth weight [14,15,16].

To prevent and manage malaria in expecting women, the World Health Organization (WHO) suggests the implementation of a comprehensive array of interventions. In areas where P. falciparum transmission is moderate to high, individuals are encouraged to utilize insecticide-treated nets, cases are promptly and effectively treated, and intermittent preventive treatment with sulfadoxine-pyrimethamine (IPTp-SP) is implemented. IPTp-SP is linked to a decrease in maternal parasitemia, a decrease in the incidence of low-birth-weight neonates, and an increase in the mean birth weight, according to data from countries with high malaria prevalence [17].

Nevertheless, IPTp therapy administered to expecting women does not ensure that their infants will be completely protected from contracting malaria. Infants born to mothers who underwent IPTp treatment are susceptible to malaria and may display a variety of symptoms. To be more specific, the primary symptoms of this condition may be diverse and frequently resemble other common neonate illnesses, such as pneumonia, meningitis/encephalitis, or gastroenteritis [4,18,19]. The sole symptoms that may be present are fever and headache, or there may be a predominance of gastrointestinal symptoms, such as diarrhea. The primary symptom is fever, but the distinct regular tertian and quartan patterns are observed in less than 25% of children. Nevertheless, children are more susceptible to experiencing elevated fevers, which can lead to febrile convulsions if they exceed 40 °C.

Furthermore, oral anti-malaria medications may be rendered ineffective by the frequent occurrence of nausea and vomiting. The most prevalent comorbidities associated with malaria are acute diarrhea and pneumonia, which are significant predictors of mortality. A concomitant bacterial or viral respiratory disease, such as pneumonia, may be diagnosed in a neonate with malaria. Additionally, a child who is experiencing respiratory distress due to malaria may be diagnosed with pneumonia. In the same vein, the presence of severe diarrhea may suggest clinical malaria or be the result of a concurrent infection with a diarrheal pathogen [20,21].

Artemisinin-based combination therapy (ACT) is employed to treat malaria in infants. Infants experience elevated rates of malaria morbidity and mortality in regions with an endemic malaria problem [22,23]. Consequently, the WHO advises the use of ACT for the treatment of uncomplicated malaria. To facilitate the treatment of minors, pediatric formulations of ACT have been created. The primary objective of pediatric malaria treatment is to administer the first-line formula of ACT, which is comprised of artemether + lumefantrine, artesunate + amodiaquine, artesunate + mefloquine, artesunate + sulfadoxine-pyrimethamine, and dihydroartemisinin + piperaquine. However, the treatment described in the formula is not the sole effective method. Another formula is the combination of oral rehydration solution (ORS). The global standard for diarrhea treatment is ORS. In its most basic form, ORS is a combination of sodium, sugar, and water that can expedite the body’s fluid replacement process [24]. ORS is administered to neonates who are experiencing diarrhea, which is one of the most prevalent symptoms of malaria in infants. Lastly, there are a limited number of studies that have assessed the influence of treatments on malaria in infants. Consequently, the current study is unique in that it evaluates the influence of the most prevalent symptoms and their respective remedies on malaria in neonates born to mothers who received three or more dosages of IPTp therapy [7,25].

## 3. Materials and Methods

### 3.1. Data

To evaluate the symptoms of malaria in infants, despite their mothers having received three doses of IPT therapy, as well as the treatment that the healthcare provider gave them, we have considered six criteria (Table 1).

**Figure 1:**
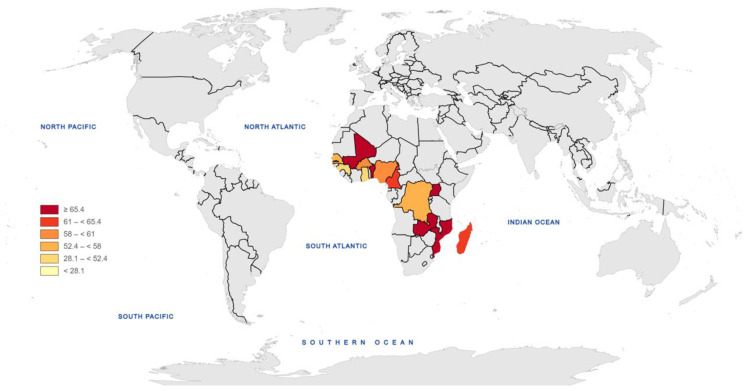
IPTp for pregnant women (aged 14–49) who received three or more doses for the year 2023.

Also, the selected variables and data were retrieved from the database of UNICEF. The data referred to fourteen African countries (Figure 1) for the year 2023. Among those, Mali, Mozambique, and Benin are the countries with the highest rate of pregnant women who have received IPTp therapy and whose infants contracted malaria.

### 3.2. Entropy Weight Method

The TOPSIS method is currently being implemented in the current research as a multicriteria decision-making approach. This approach entails the evaluation of multiple elements within a well-defined framework in relation to the data analysis method. Entropy weighing is the process of assigning weights to a diverse array of variables or factors according to the degree of uncertainty or unpredictability, as determined by entropy. Moreover, the TOPSIS model, a composite methodology that integrates entropy and TOPSIS approaches, was introduced by Kaur et al. (2023) [26]. The entropy weight approach is the primary objective of this system, which is to assess the significance of each assessment indicator. It then utilizes the method of selecting the most optimal solution to rank as-assessment items.

According to Dwivedi et al. (2023), the basic concept of the entropy weight the TOPSIS technique aims to identify the ideal solution by comparing the attribute values of several alternatives and selecting the one that has either the greatest or lowest value for each characteristic [27]. An evaluation object’s optimality is evaluated by analyzing its relative closeness to the best and worst options. Assessment items are deemed ideal when they are positioned near the most efficient solution and furthest away from the least desirable choice. On the other hand, it is deemed less than ideal if it does not match these characteristics. Entropy plays a crucial part in the TOPSIS approach by incorporating information from the original data without placing any limitations on the sample size. It offers a wide range of functions and has minimum loss of information.

The initial presumption that there are available approaches is made prior to the presentation of n alternatives $A=\{\;A_{1},\;A_{2},\;A_{3},\ldots ,\;A_{n}\}$ and *m* criteria $M=\{\;M_{1},\;M_{2},\;M_{3},\ldots ,\;M_{m}\}$, where i∈A, j∈M, i={1, 2, 3,…, n}, j={1, 2, 3,…, m}. The matrix $X^{\prime }=({x^{\prime \prime }}_{ij})$ in Equation (1) is a decision matrix of n×m. The relative importance of the criterion A weight vector can be used to represent M as follows: $W=\left\{w_{1},\;w_{2},\;w_{3},\ldots ,\;w_{m}\right\}$, which satisfies $\sum_{j=1}^{m}w_{j}=1.$

(1) $$X^{\prime }=\left[\begin{array}{ccc} {x^{\prime }}_{11} & {x^{\prime }}_{12} & {x^{\prime }}_{1m}\\ {x^{\prime }}_{21} & \ldots & {x^{\prime }}_{2m}\\ {x^{\prime }}_{n1} & {x^{\prime }}_{n2} & {x^{\prime }}_{nm} \end{array}\right]$$

The weight is determined by assessing the degree of data dispersion in the Shannon entropy weighting approach. Initially, we employ the min–max method to standardize the n original options. The subsequent step involves adjusting the standardized equation to the right by 0.001 units to facilitate future logarithmic calculations.

(2) $$x_{ij}=\frac{{x^{\prime }}_{ij}-\min\left({x^{\prime }}_{j}\right)}{\max\left({x^{\prime }}_{j}\right)-\min\left({x^{\prime }}_{j}\right)}+0.001,\;where\;i=1,\;2,\;3,\ldots ,n,\;and\;j=1,\;2,\;3,\ldots ,\;m.$$

Equation (4) was used to calculate the entropy value, represented as $e_{j}$. Entropy is a measure of the extent to which data are distributed. The entropy value drops as the data exhibit more fluctuation, indicating a higher level of information in the data. As the data become more concentrated, the entropy value rises, which suggests a reduction in the amount of information present in the data.

(3) $$r_{ij}=\frac{x_{ij}}{\sum_{i=1}^{n}x_{ij}},\;i=1,\;2,\;3,\ldots ,\;n,\;and\;j=1,\;2,\;3,\ldots ,\;m.$$

(4) $$e_{j}=-\frac{1}{\ln n}\overset{n}{\underset{i=1}{\sum }}r_{ij},\;i=1,\;2,\;3,\ldots ,\;n\;and\;j=1,\;2,\;3,\ldots ,\;m.$$

The weight $w_{j}$ is determined by Equation (5).

(5) $$w_{j}=\frac{1-e_{j}}{\sum_{j=1}^{m}\left(1-e_{j}\right)}$$

### 3.3. TOPSIS Method

In 1981, Hwang and Yoon devised the TOPSIS methodology, which quantifies the alternatives’ proximity to the optimal solutions. To ascertain the degree of proximity, we compute the Euclidean distance between each potential option and both the optimal and suboptimal alternatives. The most advantageous value for each evaluation criterion is chosen to determine the optimal solution, while the least beneficial value for each assessment criterion is used to characterize the suboptimal solution. Finally, we select the most advantageous alternative, which closely resembles the ideal resolution and significantly deviates from the unfavorable option, as the preferred decision. At the outset, we implemented individual standardization of the positive and negative attributes of the choice matrix in Equation (1) to reduce any disparities in dimensions among the various criteria. In numerous domains, we underscored the necessity of attaining uniformity through application of the min–max methodology. When determining the entropy weight, this method facilitates the process of assessing the advantages and disadvantages. A technique for order preference by similarity to the ideal solution is denoted by the acronym TOPSIS.

$$positive:x_{ij}^{+}=\frac{{x^{\prime }}_{ij}-\min\left({x^{\prime }}_{j}\right)}{\max\left({x^{\prime }}_{j}\right)-\min\left({x^{\prime }}_{j}\right)}$$

(6) $$negative:x_{ij}^{-}=\frac{\max\left({x^{\prime }}_{j}\right)-{x^{\prime }}_{ij}}{\max\left({x^{\prime }}_{j}\right)-\min in\left({x^{\prime }}_{j}\right)}$$

$$\min\left({x^{\prime }}_{j}\right)=\{\underset{i}{\min}\;{x^{\prime }}_{ij}|1<i<n,1<j<m\}$$

$$\max\left({x^{\prime }}_{j}\right)=\{\underset{i}{\max}\;{x^{\prime }}_{ij}|1<i<n,1<j<m\}$$

As illustrated in Equation (7), the dimensionless normalized decision matrix $x_{ij}$ is generated by normalizing the positive and negative criteria to generate the initial choice matrix in Equation (6).

(7) $$X^{\prime }=\left[\begin{array}{cccc} x_{11} & x_{12} & \cdots & x_{1m}\\ x_{21} & \cdots & \cdots & x_{2m}\\ \vdots & \cdots & \cdots & \vdots \\ x_{n1} & x_{n2} & \cdots & x_{nm} \end{array}\;\right],\;where\;i=1,\;2,\;3,\ldots ,\;n\;and\;j=1,\;2,\;3,\ldots ,\;m.$$

Moreover, the decision matrix in Equation (8) is obtained by multiplying each individual element $v_{ij}=w_{j}\times x_{ij},$ where $w_{j}=\left(w_{1},\;w_{2},\;w_{3},\ldots ,\;w_{m}\right)$ is obtained from Equation (5) and meets the condition $\sum_{j=1}^{m}w_{j}=1$ and $x_{ij}$, as generated using Equation (7).

(8) $$V=\left[\begin{array}{cccc} v_{11} & v_{12} & \cdots & v_{1m}\\ v_{21} & \cdots & \cdots & v_{2m}\\ \vdots & \cdots & \cdots & \vdots \\ v_{n1} & v_{n2} & \cdots & v_{nm} \end{array}\;\right]=\left[\begin{array}{cccc} w_{1}x_{11} & w_{2}x_{12} & \cdots & w_{m}x_{1m}\\ w_{1}x_{21} & w_{2}x_{22} & \cdots & w_{m}x_{2m}\\ \vdots & \cdots & \cdots & \vdots \\ w_{1}x_{n1} & w_{2}x_{n2} & \cdots & w_{m}x_{nm} \end{array}\right]$$

The positive ideal solution (PIS) is the highest value, and the negative ideal solution (NIS) is the lowest value for each criterion, as defined by Equation (9). The distance of each option from the PIS and NIS is determined using Equations (10) and (11).

(9) $$PIS:P^{+}=\left\{v_{1}^{+},v_{2}^{+},v_{3}^{+},\ldots ,\;v_{m}^{+}\right\}=\{(\underset{i}{\max}\;v_{ij}|j\in M)\}\;NIS:P^{-}=\left\{v_{1}^{-},v_{2}^{-},v_{3}^{-},\ldots ,\;v_{m}^{-}\right\}=\{(\underset{i}{\min}\;v_{ij}|j\in M)\}$$

(10) $$d_{i}^{+}=\sqrt{\overset{m}{\underset{j=1}{\sum }}(v_{ij}-v_{j}^{+}}{)}^{2},\;i=1,\;2,\;3,\ldots ,\;n,\;and\;j=1,\;2,\;3,\ldots ,\;m.$$

(11) $$d_{i}^{-}=\sqrt{\overset{m}{\underset{j=1}{\sum }}(v_{ij}-v_{j}^{-}}{)}^{2},\;i=1,\;2,\;3,\ldots ,\;n,\;and\;j=1,\;2,\;3,\ldots ,\;m.$$

Below is the computation for the coefficient of relative proximity (RC).

(12) $$RC_{i}=\frac{d_{i}^{-}}{d_{i}^{-}+d_{i}^{+}},\;i=1,\;2,\;3,\ldots ,\;n$$

## 4. Results

The research analysis was performed using the predefined choice matrix, which was divided into three major areas. The ensuing sections provide an overview of the stages of various times. The data in the decision matrix were first normalized using the N1 method. The aim of this strategy was to homogenize the data to simplify comparisons. After completing Step 1, we created a normalized matrix for each Ni. The normalized matrices may be found in Table A1 in Appendix A. In addition, the next step of the analysis included assigning weights to each criterion and creating a weighted normalized matrix using the methods described in Section 3.1 of the present research. This experiment used the entropy-weighted TOPSIS approach. Table 2 provides a comprehensive examination of the weights applied to each chosen criterion.

Subsequently, the Euclidean distance between the positive ideal solution (PIS) and the negative ideal solution (NIS) was computed. Equations (10) and (11) yielded the normalized Euclidean distance for both positive and negative solutions for each option. Table 3 presents the results.

By employing the entropy-weight TOPSIS method, it was demonstrated that criterion four (C4: infants with diarrhea and treatment with ORS and zinc) and criterion five (C5: infants with diarrhea and treatment with zinc supplements) are of the most significant importance to mothers who have received IPTp therapy to prevent malaria. The results above indicate that, even though mothers in the selected African countries had received IPTp therapy, their neonates appear to be suffering from malaria. Health providers have decided to administer a treatment that consists of a combination of ORS and zinc. Zinc supplementation has been demonstrated to decrease morbidity from infectious diseases in these populations, particularly through morbidity reductions from respiratory infections and diarrhea. In addition, the combination of zinc and ORS can be beneficial in fortifying the immune system of neonates with malaria. This therapy may prevent diarrhea episodes for a period of up to three months and expedite the recovery process. However, ORS is accessible to fewer than half of children in Africa. Consequently, the World Health Organization must incorporate co-packaged ORS and zinc into its Essential Medicines List. This will encourage African countries to prioritize the co-pack in their procurement and supply, healthcare provider training, and expenditures. Additionally, this therapy will alleviate the symptoms of malaria in neonates. Additionally, our analysis showed that one of the most common therapies for malaria in infants (C6: malaria first-line treatment for febrile infants receiving ACT) was not widespread in the selected African countries assessed in the current study.

## 5. Discussion

Neonatal malaria refers to the diagnosis of malaria in infants within the first 28 days of life. In a recent meta-analysis by Danwang et al., it was shown that the overall prevalence of clinical newborn malaria among the 28,083 neonates included was 12.0% between the 7th and 28th days of life, despite the presence of substantial heterogeneity [28]. Congenital malaria is caused by passage of malaria parasites through the placenta before birth. Neonatal parasitemia is often characterized by the presence of asexual parasites in the cord blood or peripheral blood of newborns during the first week of life. Light microscopy has proven the occurrence of clinical congenital malaria; however, the reported incidence varies significantly depending on factors such as the characteristics of the research population, the seasonal patterns of malaria prevalence, and the study technique [25,29]. The frequency of congenital malaria transmission in African nations with significant is 46.7%.

Furthermore, infants are relatively protected from malaria during their first months of life; however, infection can occur at any age and can progress to febrile illness and anemia. Infants over the age of six months are at an increased risk since they have not yet developed partial immunity, as well as the fact that their maternal antibodies and embryonic hemoglobin are diminishing [30]. Consequently, it is essential that infants receive sufficient antimalarial treatment. Additionally, IPTp therapy offers an additional layer of protection by administering antimalarial medication to expecting women at predetermined intervals, regardless of their malaria infection status. The goal is to reduce the negative consequences of a malaria infection on the mother and her embryo during pregnancy, and on the neonate. The disparities in ANC access are the subject of a new recommendation in the updated WHO guidance on IPTp. Access to IPTp can be influenced by sociodemographic factors, including age, marital status, religion, and urban/rural domicile. The adoption of IPTp is significantly influenced by socioeconomic factors, such as education, employment, and wealth index, as well as health system barriers [31,32].

The objective of the present investigation was to evaluate the most prevalent symptoms of neonatal malaria in fourteen African countries where mothers receive IPTp therapy during pregnancy. The most effective therapies for infants with malaria are also identified in this research, in addition to concentrating on symptoms. Fever is the most prevalent clinical symptom of malaria in neonates, as indicated by the existing literature (88–100%). Respiratory distress (20–57%), pallor and anemia (38% each), hepatomegaly (31–80%), refusal to feed (40–70%), jaundice, and diarrhea (25% each) are additional manifestations. Nevertheless, the present investigation indicates that diarrhea is the most prevalent symptom in neonates whose mothers have received IPTp treatment, rendering them susceptible to malaria [33]. Additionally, the combination of zinc and ORS therapy reduces the duration and severity of neonatal diarrhea, as well as its frequency over the subsequent 2–3 months. Although it is possible to describe access to treatment for neonatal diarrheal disease caused by malaria among infants as unequal, the reason for this unequal access is linked to age group, area of residence, and wealth index quintiles, particularly in countries such as those included in the current analysis. Additionally, the study’s results have the potential to disrupt the cycle of unsuccessful first-line malaria treatment. Throughout the recent past, malaria medicines have been repeatedly compromised by antimicrobial resistance (AMR). In the past two decades, we have made substantial strides in the prevention and treatment of malaria by enhancing the implementation of a variety of control measures, such as diagnostics, insecticide-treated bed nets, and artemisinin-based medications [34,35]. However, antimalarial remedies become less effective as malaria parasites develop the ability to elude their effects over time. Antimalarial drugs may require additional time to eliminate parasites when this occurs, a phenomenon referred to as partial antimalarial resistance.

Although zinc is crucial for cellular growth, differentiation, and metabolism, zinc deficiency is associated with a reduction in infection resistance and a decline in juvenile growth. Mild to moderate zinc deficiency may be prevalent worldwide, despite the rarity of severe zinc deficiency in humans. Zinc supplementation has been shown to reduce the duration and severity of diarrhea and prevent subsequent episodes, even though the mechanisms by which it exerts its anti-diarrheal effect are still unclear. Nevertheless, the management of malaria-induced pediatric diarrhea should be improved by incorporating zinc supplements, rehydration therapy with ORS, and counseling for ongoing nutrition and prevention. Lastly, policymakers should prioritize the enhancement of first-line treatment for neonatal malaria. For decades, artemisinin-based combination therapy (ACT) has been the primary treatment for malaria. Healthcare providers are no longer prioritizing it as their primary treatment option, according to the research. It is possible that this is due to the existence of potential resistance. A search for potential alternatives to ACT has been initiated in response to the emerging prospect of resistance. Therefore, future research should focus on improvement of primary treatments for neonatal malaria and integration of this therapy with zinc and ORS combination therapy.

## 6. Conclusions

Health systems in sub-Saharan Africa (SSA) have consistently acknowledged malaria as a critical public health issue, and it continues to be a primary contributor to maternal and neonatal morbidity and mortality in the region. In most sub-Saharan African countries, the utilization of insecticide-treated nets (ITNs) and antimalarial intermittent preventive therapy in pregnancy (IPTp) has experienced a substantial increase. Strategic initiatives and robust political commitments by national and international organizations have led to this result. Consequently, the incidence of malaria has decreased. Malaria in pregnancy (MiP) remains a significant preventable factor in maternal and neonatal morbidity and mortality, with an estimated 75,000 to 200,000 neonates dying in endemic regions. An expanding body of evidence supports this assertion.

Even though women receive IPTp treatment during pregnancy, the objective of this investigation was to evaluate the most prevalent symptoms of malaria in infants in fourteen African countries. The study’s results indicate that diarrhea is the most prevalent symptom in infants whose mothers have undergone IPTp therapy, rendering them susceptible to malaria. The combination of zinc and ORS treatment reduces the duration, intensity, and frequency of neonatal gastroenteritis during the subsequent 2–3 months. Infants suffering from malaria-induced diarrhea face unequal access to treatment, primarily due to factors related to age group, geographic location, and wealth index quintiles, especially in the countries under review.

Moreover, the findings of this research may call into question the effectiveness of first-line malaria treatment. Antimicrobial resistance (AMR) has increasingly undermined malaria therapies in recent decades. Over the last two decades, the improved use of several control measures such as diagnostics, insecticide-treated bed nets, and artemisinin-based medicines has achieved significant progress in the prevention and treatment of malaria. The effectiveness of antimalarial therapies diminishes when malaria parasites acquire the capacity to circumvent their effects over time. Partial antimalarial resistance may necessitate a longer duration of antimalarial drugs to eradicate the parasites. Future research proposals must target the improvement of first-line therapy for newborn malaria and advance clinical treatment. Considering the possibility of resistance, we have started research to investigate possible alternatives to ACT. Consequently, future research should concentrate on enhancing primary therapy for malaria in newborns and integrating it with a combination of zinc and oral rehydration solution (ORS).

## Figures and Tables

**Table 1:** Dependent variables and criteria with their computation units.

	Indicator ID	Indicator Description	Data Source
	IPTP	Pregnant women (15–49) who received three or more doses of intermittent preventive therapy during their previous prenatal care visits	Household surveys including MICS, DHS and other national surveys
C1	DIARCARE	Care-seeking for diarrhea for infants with malaria symptoms	Household surveys including MICS, DHS and other national surveys
C2	ORS	Treatment of gastroenteritis in neonates who were administered oral rehydration salts (ORS sachets or pre-packaged ORS fluids)	Household surveys including MICS, DHS and other national surveys
C3	ORTCF	Treatment for diarrhea in infants who have received oral rehydration therapy (oral rehydration salts, recommended homemade fluids, or increased fluids) and have continued to feed	Household surveys including MICS, DHS and other national surveys
C4	ORSZINC	Treatment of diarrhea in neonates who were administered ORS and zinc	Household surveys including MICS, DHS and other national surveys
C5	ZINC	Zinc treatment for diarrhea in neonates who have received zinc supplements	Household surveys including MICS, DHS and other national surveys
C6	MLRACT	First-line treatment for febrile infants who have previously received ACT (first-line antimalarial drug) for malaria	Household surveys including MICS, DHS and other national surveys

**Table 2:** Entropy-weight TOPSIS approach to determining criteria weight.

Criterion	C1	C2	C3	C4	C5	C6
*e_j_*	0.991	0.971	0.987	0.404	0.562	1.122
*D* = 1 − *e_j_*	0.009	0.029	0.013	0.596	0.438	−0.122
*W_j_*	0.009	0.030	0.014	0.619	0.455	−0.127

C4 > C5 > C2 > C3 > C1 > C6. Determination of the weights of the selected criteria used.

**Table 3:** Calculation of the Euclidean distance between PIS and NIS.

S_i_+	S_i−_	Si^+^ Si^−^	Si−/(Si+ Si−)
0.082385	0.095601	0.177985	0.537126
0.145773	0.0307	0.176473	0.173966
0.134343	0.040964	0.175308	0.233672
0.0309	0.168381	0.199282	0.844941
0.135921	0.053811	0.189732	0.283618
0.129315	0.047055	0.176369	0.266796
0.151772	0.028847	0.180619	0.159712
0.168552	0.023639	0.192191	0.122997
0.156242	0.025005	0.181247	0.137963
0.066952	0.108894	0.175846	0.619257
0.095928	0.077601	0.173529	0.447192
0.119909	0.055778	0.175686	0.317484
0.161518	0.023051	0.184568	0.12489
0.131546	0.048147	0.179692	0.26794

The Euclidean distances of each alternative from the PIS and NIS.

## Data Availability

Dataset available on request from the authors.

## Appendix A

**Table A1:** Normalized matrix.

C1	C2	C3	C4	C5	C6
0.0869	0.0673	0.0732	0.0318	0.0552	0.0937
0.0922	0.0581	0.0792	0.0112	0.0225	0.0899
0.0615	0.0374	0.0423	0.0158	0.0257	0.0801
0.0542	0.1404	0.0930	0.0649	0.0761	0.1109
0.0744	0.0302	0.0575	0.0116	0.0311	0.0413
0.0408	0.0406	0.0465	0.0160	0.0329	0.1032
0.0837	0.0939	0.0880	0.0124	0.0133	0.0724
0.0669	0.0949	0.0899	0.0059	0.0072	0.0717
0.0786	0.0758	0.0822	0.0080	0.0164	0.0797
0.0522	0.0715	0.0622	0.0410	0.0560	0.1035
0.0974	0.0806	0.1071	0.0327	0.0392	0.1099
0.0754	0.0925	0.0549	0.0216	0.0349	0.1228
0.0695	0.0740	0.0527	0.0078	0.0112	0.0747
0.0663	0.0428	0.0714	0.0137	0.0329	0.0797

Explaining the procedure of considering the determinants of the matrix and dividing each element by the determinants of the matrix. This approach outlines the development of the normalized matrix.
